# An integrated mixed methods approach to clarifying delivery, receipt and potential benefits of CHW-facilitated social support in a health promotion intervention

**DOI:** 10.1186/s12913-021-06778-6

**Published:** 2021-08-11

**Authors:** Maia Ingram, Kiera Coulter, Kevin Doubleday, Cynthia Espinoza, Floribella Redondo, Ada M. Wilkinson-Lee, Abby M. Lohr, Scott C. Carvajal

**Affiliations:** 1 Health Promotion Sciences College of Public Health, University of Arizona, 1295 N. Martin Ave, Tucson, AZ 85724 USA; 2 Yuma County Health Services District, 2200 W 28th St #137, Yuma, AZ 85364 USA; 3 Arizona Community Health Workers Association, 424 N Christine Ave, Douglas, AZ 85607-354 USA; 4 Mexican American Studies, University of Arizona, 1110 James E. Rogers Way, Tucson, AZ 85721 USA

**Keywords:** Social support, Community health worker, Health promotion, Mixed methods, Narrative analysis, Hierarchical cluster analysis

## Abstract

**Background:**

Social support plays a critical role in physical and emotional health, making it an important component of community health worker (CHW) health promotion interventions. Different types of support operate in different ways, however, and the relationship between the nature of CHW support and the subsequent health benefit for their clients is not well understood.

**Methods:**

This paper describes an integrated mixed methods study of the emotional, informational, appraisal and tangible support CHWs provided to Latinx community members residing in three US-Mexico border communities. Using a cohort (*n* = 159) from a CHW community-based intervention, we identify and describe four clusters of social support in which participants are characterized by life situations that informed the types of social support provided by the CHW. We examine the association between each cluster and client perceptions of social support over the 6-month intervention.

**Results:**

CHWs provided emotional, appraisal, informational and tangible support depending on the needs of participants. Participants who received higher levels of emotional support from the CHW experienced the greatest post intervention increase in perceived social support.

**Conclusions:**

Study findings suggest that CHWs may be adept at providing non-directive social support based on their interaction with a client rather than a health outcome objective. Health promotion interventions should allow CHWs the flexibility to tailor provision of social support based on their assessment of client needs.

**Supplementary Information:**

The online version contains supplementary material available at 10.1186/s12913-021-06778-6.

## Background

There is a robust and complex literature on the relationship between social support and health. Feeling loved by others or being part of a social network is associated with a person’s overall physical health and emotional wellness [1, 2]. There are layers of nuance in the delivery and receipt of support, however. Individuals perceive social support differently based on who is providing it, for example, and the perception of having social support from a loved one may be more important than its actual delivery [1]. Further, there are distinct types of support that operate in different ways. Van Dam describes these as emotional (providing affirmation and nurturance), informational (giving advice and information), instrumental (providing a tangible resource) and appraisal (helping to understand a stressful situation) [3].

A plethora of studies establish the link between the benefits of social support in managing chronic disease [4]. As a mechanism for improved health outcomes, social support can contribute to a person’s ability to cope with the stress of having a chronic disease as well as with their capacity to engage in chronic disease self-management [1]. This has led to the development of health promotion interventions designed to provide social support. Disease-focused support groups, for example, have become widely accepted as a way to create supportive spaces for people to share experiences and information. Engendering social support as a health intervention strategy is a challenging proposition, however, due to the interplay between who is providing support and what kind of support they are providing. Distinguishing the function of different types of support is important given that mismatching the type of support to a person’s needs may actually have a detrimental effect [1].

A useful distinction can also be drawn between directive support and non-directive support [5]. Directive support is defined as providing positive guidance in terms of activities like goal setting. For example, medical personnel tend to provide directive support for disease management that focuses on attaining the behavior that is most advantageous to their patients’ health. Non-directive support, on the other hand, is cooperative and focused on the perspective of the recipient of the support [6]. Non-directive support is associated with increased optimism and hope and decreased depression and loneliness [7], as well as with healthy behaviors [8]. Support groups can function as a source of non-directive support because they create a network of peers sharing a disease experience to which a person may otherwise not have access. However, many support groups are designed to focus on sharing information rather than on providing emotional or tangible support, potentially limiting their benefit [1].

Community health workers (CHWs) can play a key role in providing non-directive social support. CHWs are front line workers with a close and trusted relationship to the communities they serve. Since the 1960s, CHWs have provided a bridge between communities and the public health and medical care delivery systems to address an array of health issues and chronic disease in particular [9]. Social support is a core role of the CHW profession in the United States, delineated by specific activities such as coaching, motivating and encouraging clients [10]. Situating CHW support within typologies of the social support literature may also be useful in informing the ways in which CHWs can leverage and target their social support activities to meet diverse community needs [11]. The objective of our study was to identify types of support provided by CHWs in a community-based health promotion intervention and explore the relationship between this support and changes in perceived social support among participants.

### Community health workers and social support

Social support is a common component of CHW-facilitated health intervention studies. Unfortunately, intervention descriptions often inadequately define what CHW social support entails or discuss how a CHW determines the best ways to support their clients. An exception from the maternal and child health literature is a recent observational study by Gale et al. [12] that describes the specific ways in which pregnancy outreach workers in England provided emotional, appraisal, tangible, and informational support to their clients. The authors distinguish these instinctive supportive responses from what they call “synthetic support,” which is essentially the parameters drawn by a health intervention. They characterize synthetic support as non-reciprocal or uni-directional from the CHW to the client, time-bound by the length of the intervention, accountable to programmatic requirements for support provision and documentation and embedded in client social networks. The distinction between synthetic and instinctive support calls attention to the need to examine not only the pathways, but also the durability of interventions that provide temporary social support on prolonged health outcomes.

Within the arena of chronic disease, a systematic review of the role of CHWs in addressing hypertension found that CHW-facilitated instrumental and emotional social support enhanced self-management practices, which contributed to some health improvements [13]. In these studies, CHWs targeted their instrumental support to services related to blood pressure control, while the emotional support involved listening and motivating clients. A study of anti-viral treatment for HIV-positive individuals in Peru sought to better delineate the emotional and instrumental support that CHWs delivered as part of an intervention team. Muñoz et al. [14] used the term “matched support” to describe how CHWs were able to assess and tailor their support based on the evolving needs of the clients. Study participants reported improvements in perceived emotional and instrumental support, but the complexity of the study made it difficult to clarify which components of the intervention might have contributed to improved physical and emotional outcomes. Ingram et al. [15] described a community-based participatory study on the US-Mexico border in which the CHWs organically developed an array of support strategies to encourage diabetes self-management among farmworkers. Qualitative analysis of CHW documentation subsequently characterized the types of support as emotional, informational and tangible [15]. Participants reported increased perception of diabetes-related support and the delivery of both emotional and tangible support were independently associated with improved metabolic control. These studies suggest that because CHWs share lived experiences with the community members they serve, they may instinctively understand what kind of support individuals need at a given time [5].

As community peers, CHWs may also be skilled in offering non-directive support by listening and making suggestions without prescribing solutions or expressing judgement [7]. This process of working collaboratively with clients to help them identify and pursue their aspirations has implications for improved emotional wellness for those with chronic disease [16]. CHWs may be more likely to provide non-directive support when they have the flexibility to take a holistic approach to their clients’ health. Comprehensive rather than targeted interventions may allow CHWs to help their clients navigate social and economic situations affecting their well-being, rather than focusing on behaviors associated with a disease state. In other words, CHWs will be more able to tailor their support to client needs when an intervention is designed to encourage them to use the full scope of CHW practice [17].

## Methods

In the present study, we use a mixed methods approach to describe how CHWs provided social support (specifically tangible, informational, emotional, and appraisal social support) with Latinx community members at risk for chronic disease living on the US-Mexico border. We identify and describe four clusters of social support found in this cohort and examine their association with client perceptions of social support. We use these findings to consider how CHW interventions might be enhanced through comprehensive understanding of the different types of social support and how they function in Latino adults at-risk for or with chronic disease and people with diverse needs.

### The Linking Individuals Needs to Community and Clinical Services (LINKS) CHW Intervention

The CDC-funded LINKS study was one of many projects developed within the context of a 30-year academic-community partnership centered on U.S.-Mexico border communities, chronic disease and the CHW workforce. We provide more detail on the intervention and study methods in a protocol paper [18]. The LINKS intervention was a CHW-led community-clinical linkage model, in which CHWs in federally qualified health centers (FQHCs) referred patients at risk of chronic disease to CHWs in community settings to provide social support and referrals to community resources. In terms of the “synthetic support” aspects of the intervention, the study guidelines asked CHWs to try to maintain contact with participants for a period of six months, contacting them at least monthly to offer ongoing support. In addition to an initial baseline assessment, the CHWs conducted a follow up emotional wellness survey, developed for the LINKS intervention (see supplemental file), at the three and six-month visit. CHWs contacted participants more frequently when they were providing them with additional information and referrals or to check in. They invited the participant to contact them both during and after the intervention if they had any specific needs.

As a community-based study, the parameters of the social support were also drawn by the organizations in which the CHWs worked, for example in the flexibility of the hours or authorization to conduct home visits. The intervention was client-driven in the sense that beyond the intervention survey, the participant determined the place, content, and amount of time for the conversation with the CHW. The CHWs engaged in several core roles described in the CHW Core Consensus Project, including cultural mediation among individuals and health social service systems, providing culturally appropriate health information, providing coaching and social support advocacy, and individual capacity building [10]. In addition to health promotion activities such as chronic disease education and physical activity classes, the CHWs assembled an evolving list of resources based on the needs of the participants. The community-based CHW also communicated with the clinic-based CHW to help the participants overcome barriers to accessing health and behavioral health services.

The CHWs implemented the LINKS intervention in three Arizona counties bordering Mexico and included both rural and urban communities. The three community-based CHWs, one in each county, were Mexican-born U.S. citizens and bilingual in English and Spanish. Two of the CHWs had over 10 years of experience, while the third was a medical provider from Mexico who had migrated to the U.S. and had a strong affinity to the community she served (4th author). The Arizona Community Health Worker Association (AzCHOW) coordinated a peer support network that met monthly to provide ongoing training on the CHW core competencies (5th author). The peer network discussed recruitment strategies, approaches for developing rapport with clients, how to assess client needs, and strategies to promote emotional wellness. AzCHOW created a forum through which the CHWs could discuss the challenges they faced and the CHW with more experience in one area shared experiences and suggestions with their peers. While they did not explicitly name the type of social support, they described them as they discussed the various needs of clients. One CHW, for example, described the ways she emotionally supported her clients in her breast cancer survivor group in Nogales who were experiencing significant trauma while maintaining her own mental health. The CHW with medical experience was adept at describing her interactions with clients in the project database, and she shared the ways in which she distilled hour-long conversations into a short paragraph.

### Study approach and design

The parent study for the present study uses a prospective matched observational design to evaluate the extent to which the LINKS intervention reduces chronic disease risk and promoting emotional well-being among Latinos living in three U.S.-Mexico border communities. As a practice-based study, self-selected participants included adult patients 21 years of age or older who had a chronic disease or a pre-chronic disease including pre-diabetes, glucose intolerance or diabetes, hypertension, and high cholesterol [18]. The process for evaluating the LINKS intervention was formulated within the context of a community-based participatory research partnership and consistent with the evolving and interdependent process of intervention and evaluation development [19]. The model demonstrates the ways in which intervention designs are influenced by community and organizational contexts which then contribute to evolving theoretical and measurement approaches. Bi-directional feedback results in emerging information that motivates partners to seek out additional theories and frameworks. By integrating the LINKS intervention within practice-based settings, the CHWs were able to develop their role within their organizational context, as well as to tailor services to the specific needs of their community. In the same vein, the peer network encouraged the CHWs to develop and share new strategies in response to client needs, which were incorporated into the intervention. The research partners added or enhanced measurement tools to capture this evolving process. The co-authors on this paper include two CHWs who participated in LINKS and six members of the research team.

Our mixed methods research design included concurrent collection of qualitative and quantitative data described below and convergent analysis in which the two data types are integrated in the analysis stage. Data integration can be challenging given underlying differences in basic assumptions of each approach, with quantitative methods focusing on causality and qualitative methods seeking to elucidate how an intervention works [20]. It is thus useful to clarify objectives of data integration in the initial stage [21]. Our objective was to generate findings that could contribute to the design and implementation of CHW interventions. Consistent with recommendations of Fetters et al. [20] to improve the quality of mixed methods in health services research, we integrated qualitative and quantitative methods across the study design, analysis, interpretation and reporting stages.

### Qualitative data

The project used a HIPAA-compliant Research Electronic Data Capture (REDCap) database [22] that allowed the clinic-based CHW to refer potential participants directly to the community-based CHW. We designed the database as an interactive tool for the LINKS intervention. The CHWs could message each other regarding the status of a client, for example, to help them set up a clinic appointment or discuss eligibility status for health services. They could also track referrals from the previous visit. Additionally, the database housed the emotional well-being survey, client referrals and CHW documentation of her services. The research partners asked the CHWs to document each meeting with a client by focusing on the client’s state of mind, their current concerns and needs, and the steps they took to resolve them. The project manager trained the CHWs on how to use the REDCap database and made modifications to make it more useful based on CHW feedback. After the final intervention follow-up meeting with the client, the CHW often wrote a summary of the client’s experience with LINKS, what resources they accessed and how they seemed to have benefited. The extensive CHW documentation resulted in a series of detailed narratives of the CHW-client interaction over an approximately six-month period. While written from the CHW perspective, the descriptive nature of the narratives provides information on the delivery and receipt of different types of social support.

### Quantitative data

The emotional well-being survey included scales relevant to the measurement of social support. We used the social support index (SSI), a 7-item scale measuring perceptions of social support [23]. Items 1–6 were scored based on the participant’s identification with the item statement and item 7 was a binary response. Two of the items are as follows: Item 1 reads as “Is there someone available to you whom you can count on to listen to you when you need to talk?”, and Item 7 reads as “Are you currently married or living with a partner?” Responses for items 1–6 were coded as: 1 = “All the time”, 2 = “Most of the time”, 3 = “Some of the time”, 4 = “A little of the time”, and 5 = “None of the time.” Item 7 is coded as 2 = “No” and 4 = “Yes.” SSI scores were calculated by summing responses from the 7 items, resulting in SSI scores ranging from 8 to 34 with higher scores meaning more social support.

### Data analysis

**Fig. 1:**
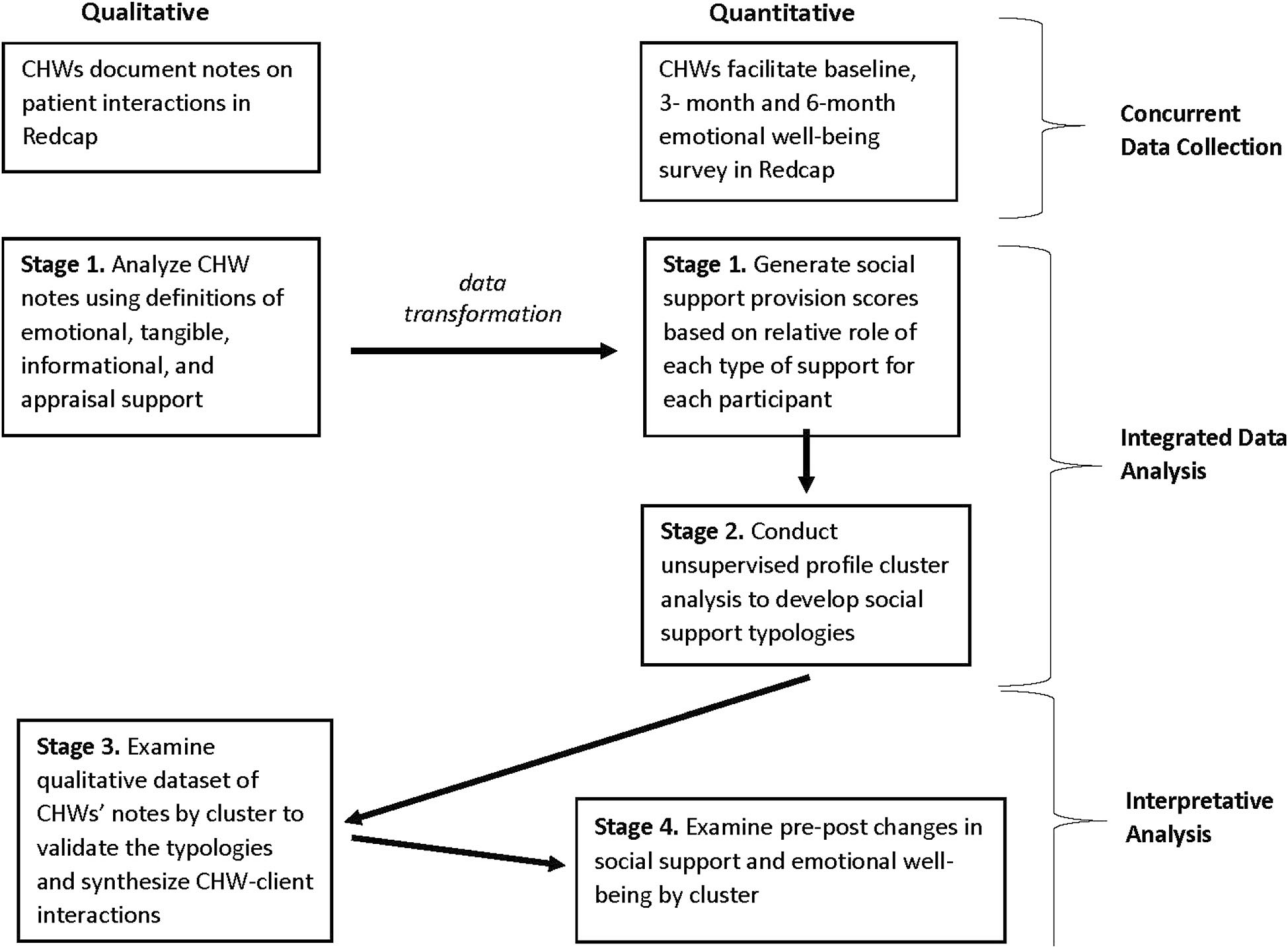
Flow Diagram of Mixed Methods

Our process of analyzing and merging the quantitative and qualitative data was iterative and occurred in several stages with each stage informing the data analysis in the next (Fig. 1). We analyzed the CHW narratives as separate strands of data to describe the types of social support CHWs provided through the intervention, as well as to transform the qualitative data into a quantitative format. We integrated the transformed data with the quantitative dataset. Analysis from the second stage resulted in four participant clusters which we described by referring back to the qualitative dataset and summarizing narratives of support within each cluster. Having validated the coherence of each cluster within the qualitative data set, our final step was to examine the relationship between the social support clusters and participant-reported social support.

#### Stage 1

We analyzed 159 narratives (1st and 2nd author). CHWs wrote, on average, 5.97 entries, ranging between two and seven entries, documenting their interactions with participants over the course of the intervention. We developed a codebook using definitions of emotional, tangible, informational and appraisal social support to code a sample of the narratives. We then divided a total of 10 points to each narrative across the four domains of social support based on our perception of the *relative* role of each kind of support over the course of the CHW-participant relationship. In assigning points, we used a holistic approach to view the entirety of the interactions and make a judgement regarding which types of support was the most salient with each client. The coders met several times with the two CHW authors to validate and refine the coding, the code definitions and their concurrence with how we had distributed the 10 points. After finalizing the codebook, we double coded and assigned points to one-third of the narratives across the three CHWs. We then compared the point distribution, using color-coding to reflect and discuss the rationale when differences occurred. There were 17 of the participants with discrepant social support provision scores, which the authors reconciled through direct discussion. Finally, having reached a comfortable level of agreement we separately coded the remaining narratives.

#### Stage 2

We used heat map visualization (3rd author) to explore the distribution of social support scores among the four domains. We then examined the distribution of social support across all participants and subgroup specific summaries for gender and age. We clustered LINKS participants according to social support domain scores using a hierarchical agglomerative clustering algorithm. We opted to use Ward’s method to define the clusters [24]. Briefly,

1. Each observation is placed in its own cluster to start, i.e. begin with *n* distinct clusters each with a single observation.
2. Create *n-1* clusters by considering all pairs of clusters and identifying the pair of clusters that are “most similar”. Ward’s clustering method assesses similarity by error sum of squares.

Let ${x}_{ijk}$ denote the observed value of the ${j}^{th}$ variable for the ${i}^{th}$ observation from the ${k}^{th}$ cluster. In the first iteration of the algorithm we have $k=n$. The error sum of squares corresponding to merging clusters ${k}_{1}$ and ${k}_{2}$ is defined as,

$$ ES{S}_{k_1,{k}_2}={\sum}_i{\sum}_j\left({\left({x}_{ij\left({k}_1,{k}_2\right)}-{\overline{x}}_{i\cdot \left({k}_1,{k}_2\right)}\right)}^2+{\left({x}_{ij\left(k`\right)}-{\overline{x}}_{i\cdot \left(k`\right)}\right)}^2\right) $$

where ${x}_{ij\left({k}_{1},{k}_{2}\right)}$ denotes observations in either cluster ${k}_{1}$ or ${k}_{2}$ and ${x}_{ij\left(k`\right)}$ denotes observations NOT in cluster ${k}_{1}$ or ${k}_{2}$. The quantity ${\overline{x}}_{i\cdot \left({k}_{1},{k}_{2}\right)}$ corresponds to the mean for variable *j* when observations in clusters ${k}_{1}$ and ${k}_{2}$ are combined. The quantity ${\overline{x}}_{i\cdot \left(k`\right)}$ corresponds to the mean for variable *j* for observations not in clusters ${k}_{1}$ or ${k}_{2}$.

Generally, the sum of squared error can be expressed for a given set of *k* clusters as,

$$ESS={\sum }_{i}^{}{\sum }_{j}^{}{\sum }_{k}^{}{\left({x}_{ijk}-{\overline{x}}_{i\cdot k}\right)}^{2}$$

(3) All pairwise cluster ESS estimates are compared and the pair of clusters yielding the smallest ESS estimate are combined to form a new cluster.
(4) Steps 2 and 3 are repeated until all observations are contained in a single cluster.

**Fig. 2:**
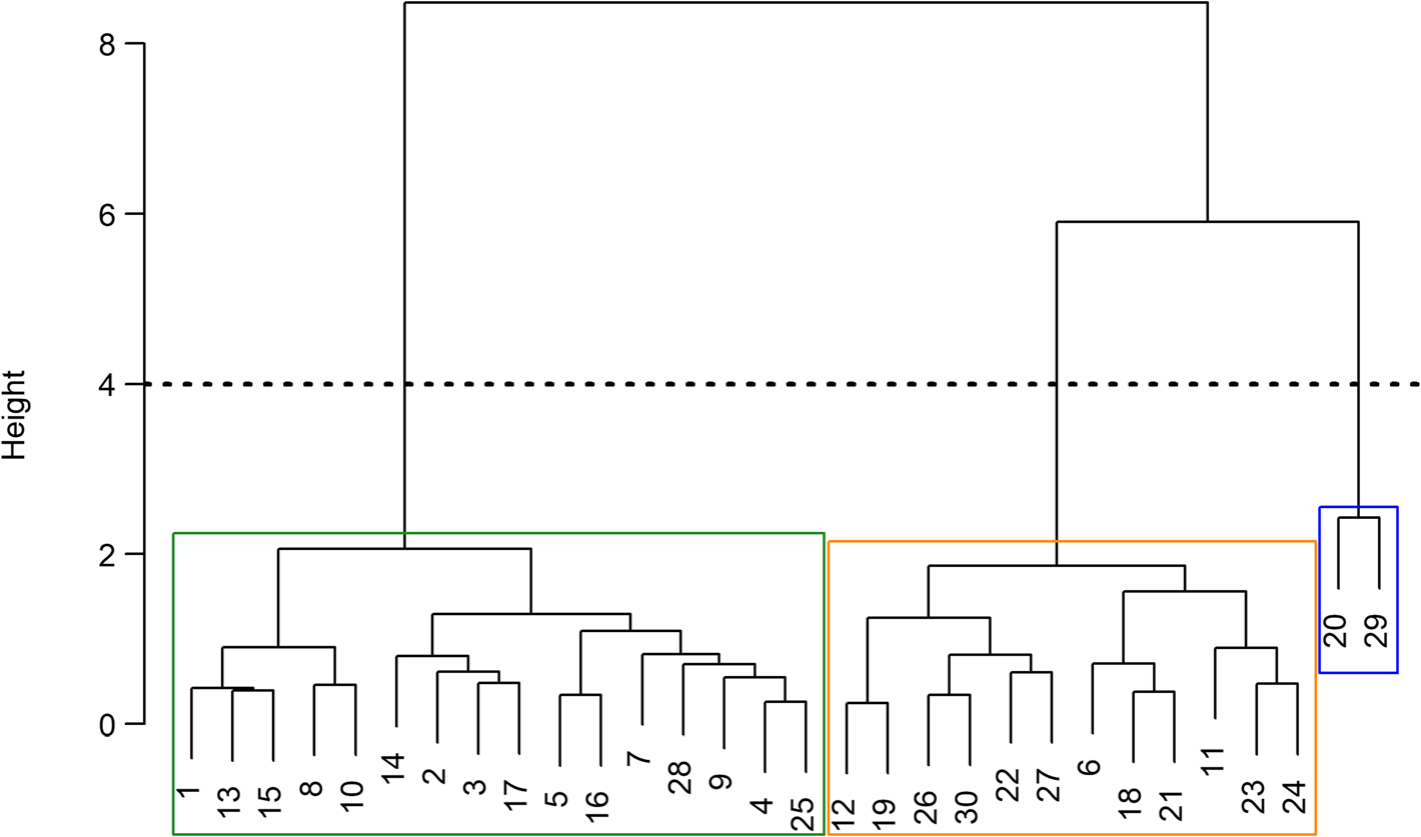
Example Dendogram for hierarchical clustering

This hierarchical clustering strategy can be represented in a dendrogram. Figure 2 provides an example of a dendrogram summary of a hierarchical clustering procedure. Each observation falls into a single bin at the bottom of the figure. The y-axis corresponds to the height, or distance, between clusters. Observations with a greater degree of similarity appear closer in the dendrogram tree structure. On the far right side of the figure, observation 20 is highly similar to observation 29 since they share a common branch. Cluster designations for each observation can be obtained via a cut made in the tree structure. The dotted line in Fig. 2 is an example of such a cut that results in three clusters, which are annotated with colored boxes.

#### Stage 3

We (1st and 2nd author) created a file that delineated the narratives associated with each cluster. We reviewed each narrative and made notes on CHWs' observations of the characteristics of the participant, the information participants shared with the CHW and the ways in which participants utilized and seemed to benefit from CHW support. We summarized our overall impression of each cluster separately and then compared notes to validate our impression that each cluster represented a group of participants with comparable situations and social support needs. We subsequently met with the two CHW authors to further validate and describe each cluster.

#### Stage 4

Finally, in order to explore the relationship between the social support clusters and social support outcomes, we summarized the mean change in the SSI from baseline to 6-month follow up within each cluster.

## Results

The CHWs enrolled 189 participants into the LINKS intervention; Table 1 presents the baseline characteristics of the LINKS participants. Of these, 159 (84 %) participants had at least two CHW contacts over the course of the six-month intervention. The majority of participants identified as Latinx (social support analysis group: 96.2 %; Excluded: 86.7 %; *p* = 0.055). A majority in both groups were female (social support analysis group: 88.1 %; Excluded: 73.3 %; *p* = 0.046). Participants in the social support analysis group were on average 56 years old (SD = 14) compared to 60.1 years (SD = 13.5) among those excluded from analysis (*p* = 0.143). Social support inventory (SSI) scores at baseline were similar in both groups [social support analysis group mean = 27 (SD = 5.9); Excluded: mean = 25.9 (SD = 7.4); *p* = 0.427]. The internal consistency for the SSI scale was high at baseline [Cronbach’s $\alpha$ = 0.81; 95 % CI: (0.76, 0.85)] and at 6 months follow up [Cronbach’s $\alpha$ = 0.75; 95 % CI: (0.68, 0.81)].

**Table. 1:** Baseline characteristics for LINKS participants stratified by meeting social support analysis group inclusion criteria

Characteristic	Social Support Analysis Group (*n* = 159)	Excluded from Analysis (*n* = 30)	p*
Sex, Female, n (%)	140 (88.1)	22 (73.3)	0.046
Age, mean (SD)	56.0 (14.0)	60.1 (13.5)	0.143
Ethnicity, Latino/a, n (%)	153 (96.2)	26 (86.7)	0.055
SSI Score, mean (SD)	27.0 (5.9)	25.9 (7.4)	0.427

*p-values are from Fisher’s exact test for sex and ethnicity and two sample t-test otherwise

### Social support domains

**Fig. 3:**
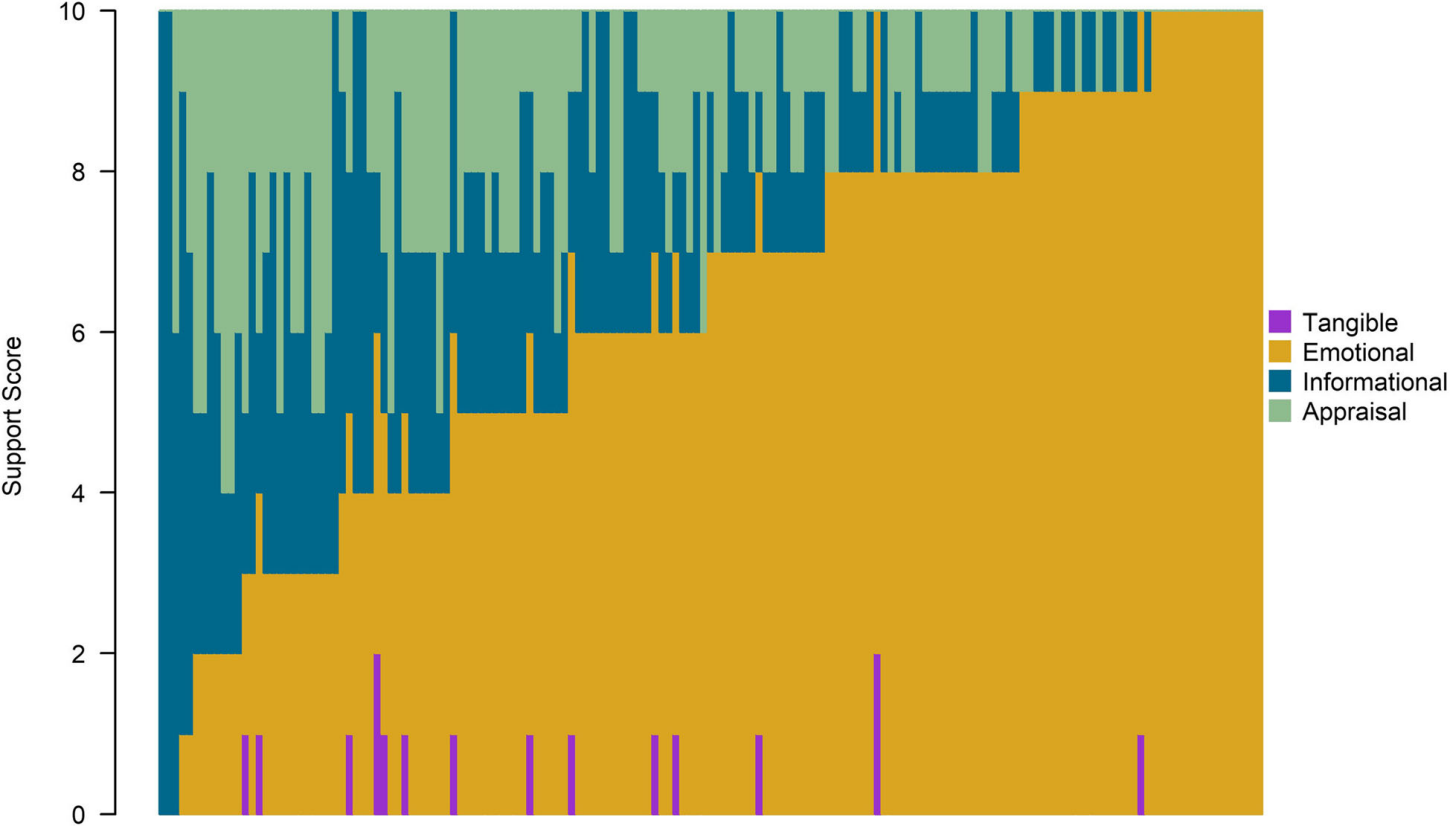
Distribution of Social Support Domains

Figure 3 displays the distribution of the scores for social support domains. Each participant occupies a vertical slice of the plot. Each slice is divided up into 10 units corresponding to the social support domain score assigned to the participant. The four domains correspond to a color representative (tangible = purple; emotional = yellow; informational = blue; appraisal = green). Across the entire LINKS cohort, the mean emotional support score was 6.7 (SD = 2.6), the mean informational support score was 2.0 (SD = 1.9), the mean appraisal support score was 1.6 (SD = 1.5), and the mean tangible support score was 0.1 (SD = 0.3). Hence, CHWs provided emotional support in roughly a 2:1 ratio compared to all other domains of support. CHWs provided the least amount of tangible support across the entire cohort (roughly 1:100 ratio to other domains).

### Cluster analysis

We identified four clusters using Ward’s hierarchical clustering method. Table 2 presents the social support domain summaries across cluster. Cluster 1 contains 30 participants and corresponds to high levels of emotional support provided (mean emotional support 9.5) with minimal informational (mean 0.4) and tangible support (mean 0.1). Cluster 2 contains 67 participants corresponding to high emotional support (mean 7.3) and a small level of informational (mean 1.6) and appraisal (mean 1.0) support. Cluster 3 contains 15 participants who received higher levels of informational support (mean 6.3) along with a small amount of emotional (mean 2.3) and appraisal (mean 1.3) support. Cluster 4 contains 47 participants who received a balance between emotional (mean 4.1), appraisal (mean 3.4), and informational (mean 2.3) support.

**Table 2:** Clustering Results by Social Support Domain *N*=159

Cluster (n)	Social Support Domain (Mean Score)			
	Emotional	Tangible	Appraisal	Informational
1 (*n*=30)	9.50	0.10	0.00	0.40
2 (*n*=67)	7.27	0.09	1.03	1.61
3 (*n*=15)	2.33	0.00	1.33	6.33
4 (*n*=47)	4.11	0.15	3.45	2.30

### Cluster narratives

A review of the cluster narratives revealed common characteristics of the individuals, their life situations and provision of support during their interaction with the CHWs. Table 3 provides a sample of the narratives making up each cluster.

**Table 3:** Cluster Narrative Examples

Cluster 1: High emotional, minimal tangible & informational	The participant had diabetes. She describes herself as a very active woman who wanted to be self-sufficient. She said that her only fear is having to depend on someone else. She was careful to follow a routine to keep her diabetes under control. She expressed interest in a diabetes class but was reluctant due to her schedule. Over the course of the six months, her routine was disrupted when her son’s family moved in temporarily due to housing issues and the CHW talked with her extensively about how to manage this situation. The participant expressed strong appreciation for the relationship she had developed with the CHW. The participant was experiencing a recurrence in cancer at age 37 and was in treatment and suffering from the effects of radiation. The CHW talked with the participant about her concern for her 15-year-old son. The CHW provided emotional support and emphasized the client’s personal qualities, describing her strength in facing the cancer and the treatment and her ability to maintain her emotional stability while suffering the effects of treatment.
Cluster 2: High emotional, low appraisal & informational	The major issue facing this participant was the process for applying for citizenship because it required that she take the exam in English. In the first visit, the participant explained that her doctor ignored her request to provide the paperwork that would allow her take the test in Spanish. Over the course of the six months, the CHW provided ongoing emotional support, encouraging her to submit her paperwork. The anti-immigrant sentiment in the news caused the participant further anxiety and the CHW encouraged her to not allow herself to be paralyzed by fear and to have confidence in herself. The participant was providing childcare for her son, but she also wanted to find a job, and the CHW provided resources on different types of work and agencies that could help. The participant stated that she appreciated the CHW support and felt that the CHW understood her. This participant described many issues in the initial visit. Her husband had renal insufficiency and she had to take him to various doctor’s appointments. He was verbally abusive and she wanted to learn ways to deal with him. Her son was in jail in Mexico and she was worried about his welfare. She had become more isolated after her daughter left town and the CHW offered a variety of different health programs, but the participant felt too busy. The CHW offered her behavioral health resources, but the participant didn’t feel it was a priority. The CHW also provided resources for employment and financial assistance.
Cluster 3: High informational, low emotional & appraisal	The participant was very busy; she was taking care of an ailing parent and worked, making it hard to follow up with her at times. In the midst of those challenges, she did use referrals to medical assistance, hospice care and legal assistance. She showed interest in the diabetes and Tai Chi workshops but ultimately never had time. She mentioned she felt like she ran around most of the day. She said she appreciated the emotional and information support she received from the CHW. At the first visit, the participant expressed emotional need, but from then on seemed very busy with her job and unable to take advantage of the services the CHW offered. The participant was looking for a job, dealing with health issues and busy with family. It seemed the participant really wanted to learn English, engage in yoga, and complete her GED, but just could not make time to do it. The CHW kept providing resources or alternative ways of getting resources (i.e. yoga via YouTube) should she decide to move forward.
Cluster 4: Balanced emotional, appraisal & informational	The participant was interested in accessing resources and took advantage of CHW referrals to health promotion and computer classes. He was also seeking to reenter the workforce and expressed some anxiety about this. He met a woman who worked in the Mexican consulate in one of the classes who helped him develop a project he had been incubating. He expressed appreciation for the emotional support that the CHW provided over the six months. The participant had recently separated from his wife and found that he could open up to the CHW and talk about his concern that he might be feeling depressed. The CHW provided referrals for behavioral health and other resources. He engaged with the CHW at the clinic and started taking diabetes education classes, which led to behavior change and desired weight loss. The participant expressed how difficult it was for him to take about his emotions and that he was discouraged when his behavioral health counselor was changed on him.

#### Cluster 1 (High emotional, minimal tangible/informational)

Many of the participants in this cluster were managing chronic pain or other illness and the CHWs coached them through various strategies. While they sometimes expressed a desire to initiate more physical activity, their medical condition often made this difficult. Some of these participants were experiencing difficult family situations. There were participants in this group who expressed depressive symptoms or feelings of overwhelming emotional stress and appreciated CHW emotional support as they moved through their issues. A portion of these participants were doing well overall, and they enjoyed sharing stories of their activities and lives with the CHW.

#### Cluster 2 (High emotional, low appraisal/informational)

Many of these participants were dealing with emotional and situational stress and were experiencing anxiety and depressive symptoms. Participants were interested in health programs and other resources that the CHWs had to offer. Over the course of the program, the participant and CHW talked about health, with the CHWs helping in a “coaching” role through non-judgmental listening. In this role, CHWs actively listened to the participants describe stressful situations and supported clients in reflecting on their situations. When participants sometimes expressed disappointment in their progress the CHW was affirming of their effort and encouraged them to be more forgiving of themselves, and offered suggestions on how they could change negative thoughts into positive ones. CHWs continually offered services, although the clients described many barriers such as work demands, lack of transportation, or inability to afford childcare.

#### Cluster 3. (High informational, low emotional/appraisal)

The majority of these participants were very busy and fairly self-sufficient and sometimes difficult to reach. The CHWs provided resources, but the participants frequently said they had not been able to take advantage them in the time between visits. They expressed appreciation for the fact that the CHW checked in with them but the CHW did not develop a deeper connection. Some participants wanted to learn about resources to share with others.

#### Cluster 4. (Balanced emotional, appraisal and informational)

Many of these participants started out the first visit expressing a specific goal, such as the desire to improve chronic disease risk or apply for citizenship classes. The participants were busy and their needs evolved and were both concrete and complex (i.e. citizenship, employment) requiring different types of support at each visit. The CHWs animated, motivated, and encouraged participants to follow through with the services, often providing reinforcement for activities that the participants were already involved in, learning English, in particular. The CHWs provided them an opportunity to talk through their fears, thoughts and progress on their plans. There were more men in this particular cluster.

### Participant clusters and changes in perceived social support

The SSI change score represents the difference between SSI score at 6-months and baseline as shown on Table 4. There were 3, 8, 2, and 6 participants from Clusters 1–4 with missing data from the SSI questions at either baseline or 6-months follow up. There were eight participants with absolute change in SSI greater than 13. There is a notable relationship between the level of emotional support provided and the perceived change in social support. Specifically, those receiving higher relative levels of emotional support reported greater increases in perceived social support over the course of follow up. Clusters 1 and 2 received the greatest amount of emotional support from the CHWs, 9.5 and 7.3 out of 10, respectively (Table 2). These two clusters also reported the greatest increase in perceived social support [mean SSI increases of 2.6 (SD = 6.6) and 2.9 (SD = 5.5), respectively]. The Cluster 3 received mostly informational support from the CHWs (6.3 out of 10) and reported the smallest increase perceived social support of the four clusters [mean SSI increase = 1.1 (SD = 3.2)]. Cluster 4 received a balance of emotional, appraisal, and informational support from CHWs and reported a mean increase in perceived social support of 1.9 (SD = 4.4).

**Table 4:** Social Support Inventory Score by Cluster

Cluster (*n*)	SSI Baseline mean (SD)	SSI Follow Up mean (SD)	SSI Change from Baseline Mean (95% CI)	*p*
1 (*n*=27/23^a^)	26.1 (7.8)	28.7 (4.8)	+2.6 (0.0, 5.1)	0.053
2 (*n*=59/55^a^)	26.7 (5.4)	29.6 (4.6)	+2.9 (1.5, 4.3)	< 0.001
3 (*n*=13/13^a^)	28.2 (4.9)	29.3 (4.6)	+1.1 (-0.9, 3.0)	0.252
4 (*n*=41/41^a^)	26.8 (6.3)	28.7 (5.9)	+1.9 (0.5, 3.3)	0.008

^a^Individuals with SSI Score

## Discussion

The LINKS pre-post evaluation demonstrated that perceptions of social support increased among participants over the 6-month intervention. While informative, as with any assessment based on mean response, this finding fails to capture which aspects of CHW-client interaction might have contributed to changing perceptions, or to consider the variability in benefit among individual respondents. The mixed methods and integrated analysis allowed us to capitalize on the CHWs’ detailed descriptions of their interactions and explore the dynamics between CHW support delivery and client support response. Transformation of these data and cluster analysis led to the identification of four clusters of social support interaction, with a higher proportion of emotional support associated with greater changes in perceived benefit. These findings highlight the contribution of non-judgmental listening as a form of CHW social support, and specifically within the LINKS intervention, to listen without an agenda for any specific behavior change on the part of the client.

The magnitude of the perceived change in social support varied across the four clusters with greater increases associated with the clusters of participants who were provided more emotional support. Rather than interpreting this as a deficiency in other types of social support, we interpret this finding as driven by participant preferences and needs. The narratives help us to understand these differences. Those who chose to share personal struggles with the CHWs were seeking and subsequently experienced emotional support, while those in the high informational cluster were interested in resources but were less interested in or able to engage in an extensive emotionally supportive interactions. It is notable that that those in cluster 2 who received high emotional support coupled with some appraisal and informational support experienced the greatest increase in perceived social support. The narratives suggest that the individuals in this cluster described a situation or need, and that the CHWs were then able to help them frame the issue and tailor their emotional and informational support to resolve this issue. The narratives also provide some insight into why individuals in cluster 3, the high informational cluster, benefited less from the intervention in terms of perceived social support. These individuals described themselves as busy and were most interested in resources for themselves or others. They were hard to reach for follow up, and the CHW often interacted with them by phone.

### Implications for research and practice

Regarding the application of social support in CHW practice, the cluster analysis offers unique insight (or lens) to interpret our data. We considered, but ultimately rejected, the idea of an initial social support assessment serving as a tool to help CHWs tailor their social support efforts to be more efficient and perhaps effective. The CHW authors maintained that non-judgmental listening is an intrinsic quality of the CHW-client interaction and that having a preconception of their clients’ needs might color their ability to listen with an open mind. From the perspective of the CHW profession, there is no short cut to being available and persistent, continuing to offer services as client needs and circumstances shift and evolve. Findings from the cluster analysis could contribute to enhancing CHW core competency training by better preparing CHWs to recognize clients' emotional status and tailor support efforts within the context of a specific visit, as well as in ongoing relationships with clients. Further, greater awareness of social support dynamics in core competency training would also facilitate CHWs’ capacity to analyze the complexity of client needs. Given that CHWs tend to take responsibility for their ability to connect with clients, CHWs may also be buoyed by the knowledge that a client is likely to benefit from any kind of social support interaction, including those in which the client seems less receptive to emotional or appraisal support.

With respect to synthetic aspects of health promotion interventions that include social support, LINKS provided minimal guidelines beyond monthly contact and the administration of the survey to promote flexibility and CHW responsiveness to client identified needs, the content of which certainly facilitated a discussion of emotional well-being. In practice, CHW programs are often more specific in the type of services they offer and the targeted health outcomes. These guidelines may channel CHWs into interactions that favor the needs of the program over those of the client, potentially limiting the scope of social support that CHWs offer. Additionally, asking CHWs to target a specific health outcome, such as improved blood pressure, may result in CHWs offering more directive support or support with a specific behavior change agenda. Our study findings reinforce delineation of a distinct CHW role as members of health care teams to provide patients with non-directive support. Providing the autonomy to CHWs to tailor services to client needs, perhaps de-emphasizing behavioral change and health outcomes, may be challenging to health care organizations constrained by payment structures. However, innovative funding models coupled with the recent emphasis in health care to respond to the social and economic circumstances impacting health [25, 26] may provide new opportunities to incorporate this flexibility into CHW clinical roles. Community-based organizations may be more able to accommodate CHW flexibility and spontaneity in working with their clients [27]. These organizations may also be more equipped to develop a response to emerging community needs that CHWs identify in their client interactions through programmatic or policy efforts.

Our study contributes to a growing body of evidence that perception of greater social support is a prime outcome of CHW practice [28]. Incorporating measures of social support into standard evaluation of CHW programs would contribute to intentional integration of the range of social support types into training and practice [29]. It would also help to capture they ways in which CHWs administer social support, allowing for further investigation into the connection to health benefits. An important next step in increasing our understanding of CHW-facilitated social support is to associate these interactions not only to perceived social support, but also to other health and emotional outcomes.

### Limitations

Our study of social support was ambitious in its effort to balance the contribution of quantitative and qualitative methods across data collection, analysis and interpretation processes. The value of mixed methods research was evident in using analytical approaches to build upon the strengths of each method, as well as to cross-validate findings at each stage of analysis. A major limitation of our study is that we did not include randomization to a comparison condition in LINKS, creating the potential for selection bias. Additionally, while our study methods allowed us to construct clusters of social support provision, the study was not powered with constructing these clusters in mind. Rather, our findings provide evidence to justify conducting a larger scale study with these clusters explored in the primary analysis. A further limitation of our data is that we were not able to connect the social support clusters with subsequent health outcomes. However, given the large body of research regarding the health benefits of social support, the focus of this study was to better understand the role of CHWs in assessing and providing social support in a health promotion intervention. While we do not consider our findings generalizable because the intervention prioritized a population with specific needs, which may have influenced the types of support provided, the innovative mixed methods approach may be useful to a variety of contexts.

## Conclusions

Studies have established the critical role of social support in improving physical and emotional health, particularly as it relates to chronic disease management. Two aspects that influence the degree of social support benefit are who is delivering the social support and whether the social support is congruent with the recipient's needs. CHWs are ideally positioned to provide effective social support given that they able to quickly establish rapport and engender trust and have a deep understanding of the challenges and circumstances affecting community residents. We found that when given sufficient autonomy, experienced CHWs are able to recognize the different components of social support and tailor their approach to clients in a masterful way. However, the ways in which CHWs provide social support and their impact on their clients’ perceptions of social support requires more study within existing literature, which the current mixed-methods study sought to address. Our findings demonstrate that CHWs' social support is patterned after specific characteristics and needs of their clients. Study findings emphasize that CHW-facilitated health interventions give CHWs the agency to determine the provision of social support. Future research should continue examining CHW-provided social support, given growing recognition of their efficacy in health interventions, and linking CHW-provided social support to physical and emotional health outcomes.

## Supplementary Information

**Additional file 1. ** Emotional Well-being Questionnaire

## Data Availability

The data presented in this study are available on request from the corresponding author.

## Abbreviations

CHW: Community health workers
LINKS: Linking Individual Needs to Community and Clinical Services
FQHC: Federally qualified health center
